# Protection of Health Care Professionals During an Epidemic: Medical, Ethical, and Legal Ramifications

**DOI:** 10.2196/19144

**Published:** 2020-07-08

**Authors:** Achuta Guddati

**Affiliations:** 1 Augusta University Augusta, NY United States

**Keywords:** medical ethics, harm, protection, COVID-19

## Abstract

The welfare of health care professionals working in hazardous environments is a concerning issue. Personal protective equipment such as face masks, disposable gowns, hair covers, gloves, and shoe covers is often used to prevent contamination from patient contact and droplets. This is especially relevant during an epidemic, when health care professionals are at elevated risk of infection. Failure to provide adequate protection to health care workers during epidemics has medical, ethical, and legal ramifications.

## Introduction

Coronavirus disease (COVID-19) is caused by a novel coronavirus, severe acute respiratory syndrome coronavirus 2 (SARS-CoV-2); the disease manifests as severe acute respiratory illness in some patients and has been spreading globally since December 2019 [1]. Recently, a trainee physician pursuing a fellowship in sleep medicine decided to volunteer at a local hospital on the weekend to help address a physician shortage. Upon reaching the hospital, the physician was asked by the hospital administrators to help with coverage on a COVID-19 rule-out floor. He learned that the hospital system had dedicated one of its hospitals exclusively to the treatment of patients who tested positive for COVID-19, while the other hospitals in the system had each dedicated a ward to ruling out patients with COVID-19. The request to work on the COVID-19 rule-out floor was unexpected; however, recognizing the acute shortage of physicians, the physician agreed to work on this floor, where patients who were symptomatic but had uncertain history of exposure were being managed while their viral test results were pending. Upon entering the floor, the physician donned a surgical mask and proceeded to see his first patient. Before entering the room, he was stopped by the “manager” of the floor and was ordered to remove the mask. When the physician asked for the reasoning behind the order, he was told that the mask was not necessary while seeing patients who were not confirmed to be positive for COVID-19 and that the hospital needed to conserve masks. The physician disagreed with the manager and proceeded to examine the patient while wearing the mask. He explained to the patient that there was no cause for alarm and that he was wearing the mask only as a precautionary measure. The patient agreed and mentioned that it was important that the doctor did not contract the disease as well. When the physician left the room, the manager was waiting outside and insisted that the physician remove his mask because the other patients would panic if they saw all the medical staff on the floor wearing face masks. The physician disagreed and proceeded to see other patients while wearing the face mask; a few hours later, some of the other physicians on the floor started to use face masks. After completing his shift, the physician informed the medical staff that he felt that his life was endangered by the hospital policy and that would not be able to volunteer for any more shifts unless the policy was changed to allow use of face masks by physicians on the COVID-19 rule-out floor. This scenario depicts a real encounter which recently occurred and is typical of the many friction points that have been arising between health care professionals and the administrative staff of hospitals during the pandemic.

## Medical Aspects

A COVID-19 rule-out floor has a higher probability of having infected patients, and exposure to these patients without adequate protection indeed poses a grave risk for health care professionals and their families. The morbidity and mortality of health care workers due to COVID-19 infection is suspected to be higher due to their exposure to higher viral loads. Allowing health care professionals to work unprotected in such environments will also increase the likelihood of contagion of their family members. The most critical consideration that must be taken into account is that the spread of COVID-19 infection to health care professionals will likely exacerbate the shortage of available personnel. The benefits of physicians wearing face masks to shield themselves from potential carrier patients outweigh the risk that the mask will act as a medium of contamination between patients. Furthermore, wearing one mask throughout a shift is different from donning a new mask when entering a different patient’s room. However, the use of other protective wear such as gowns could lead to a rapid depletion of their supply. Hence, allowing health care workers to use face masks is a reasonable approach to prevent the spread of infection from patients to health care workers. This measure strikes a balance between using resources judiciously (one face mask vs several face masks and gowns) and protecting health care workers from infection. There is growing evidence that wearing face masks and maintaining social distance are the two most important factors in preventing the spread of COVID-19.

## Ethical Aspects

Health care workers who continue to work in medical wards and intensive care units treating patients with COVID-19 are aware that they are at higher risk of contracting the infection. Continuing to work in such situations requires a moral commitment toward patient care that goes beyond the worker’s job description. Many health care workers, especially physicians, may have sufficient financial reserves to stay away from work and go without pay until the epidemic subsides. In the absence of additional financial incentives for physicians to work in a hazardous environment, one can only rely on their consciences to motivate them to continue to contribute to patient care. Most people would agree that a physician who abstained from work due to a hazardous infectious environment would be within their legal rights; however, such an act would be considered unethical. Denying basic protective wear such as face masks to health care professionals in the current situation shows that hospital administrations are not only disregarding the altruistic nature of medical care but are themselves committing an unethical act of deliberately increasing the likelihood that health care professionals will be infected. Hospital administrations are expected to follow an ethical code of conduct while balancing the provision of patient care with the safety of the people who are providing it. It would be unimaginable to send firefighters with minimal protective gear to control raging fires, and such an act by the government would expose these personnel to grave risk of injury. This parallel is drawn between firefighters and health care professionals due to the similarity of the risks involved and the threats to human life that both professions experience.

## Legal Aspects

All employees, regardless of their profession, are entitled to a safe work environment per US Occupational Safety and Health Administration (OSHA) guidelines [2]. Lack of data about the transmissibility of COVID-19 does not constitute reasonable grounds to deny minimal protection to a health care worker, especially when they are working in a high-risk environment such as a COVID-19 rule-out floor. There have been several recent reports of health care workers who raised concerns about safety being threatened by their employers with termination of employment [3]. It is possible that in the current emergent situation of a rapidly spreading viral infection, there is little time to debate the pros and cons of occupational health hazards. However, it is clear beyond reasonable doubt that denying basic protective wear such as face masks to health care professionals who are working on medical floors with patients highly suspected of being infected with COVID-19 constitutes criminal conduct. Even if no viral infection is contracted during such patient encounters, the mental stress to which these health care professionals are subjected may form grounds for legal recourse.

## Conclusion

Health care professionals are cognizant of the acute shortage of personal protective wear in the current scenario of an epidemic. The Hippocratic Oath binds physicians to uphold ethical standards; however, a moral code of conduct is also expected from hospital administrations while balancing the provision of patient care with the safety of the people who are providing it. There will undoubtedly be much debate about this issue after this epidemic subsides, especially because there is currently a consensus that everyone should wear a face mask to decrease the possibility of infection with COVID-19. Adversity tests character—both of individuals and of organizations.

## Abbreviations

COVID-19: coronavirus disease
OSHA: Occupational Safety and Health Administration
